# A comparison of physical and psychological features of responders and non-responders to cervical facet blocks in chronic whiplash

**DOI:** 10.1186/1471-2474-14-313

**Published:** 2013-11-04

**Authors:** Ashley Dean Smith, Gwendolen Jull, Geoff Schneider, Bevan Frizzell, Robert Allen Hooper, Michele Sterling

**Affiliations:** 1 Division of Physiotherapy, NHMRC Centre of Clinical Excellence Spinal Pain, Injury and Health, University of Queensland, Brisbane, Australia; 2 Evidence Sport and Spinal Therapy, C/- The Advanced Spinal Care Centre (EFW Radiology), 201, 2000 Veteran’s Place NW, Calgary, AB T3B 4N2, Canada; 3 Faculty of Medicine, University of Calgary, Calgary, Alberta, Canada; 4 Centre of National Research on Disability and Rehabilitation Medicine, The University of Queensland, Brisbane, Australia

**Keywords:** Whiplash, Facet joint injections, Sensory hypersensitivity, Central hyperexcitability, Post traumatic stress, Psychological distress, Neck pain

## Abstract

**Background:**

Cervical facet block (FB) procedures are often used as a diagnostic precursor to radiofrequency neurotomies (RFN) in the management of chronic whiplash associated disorders (WAD). Some individuals will respond to the FB procedures and others will not respond. Such responders and non-responders provided a sample of convenience to question whether there were differences in their physical and psychological features. This information may inform future predictive studies and ultimately the clinical selection of patients for FB procedures.

**Methods:**

This cross-sectional study involved 58 individuals with chronic WAD who responded to cervical FB procedures (WAD_R); 32 who did not respond (WAD_NR) and 30 Healthy Controls (HC)s. Measures included: quantitative sensory tests (pressure; thermal pain thresholds; brachial plexus provocation test); nociceptive flexion reflex (NFR); motor function (cervical range of movement (ROM); activity of the superficial neck flexors during the cranio-cervical flexion test (CCFT). Self-reported measures were gained from the following questionnaires: neuropathic pain (s-LANSS); psychological distress (General Health Questionnaire-28), post-traumatic stress (PDS) and pain catastrophization (PCS). Individuals with chronic whiplash attended the laboratory once the effects of the blocks had abated and symptoms had returned.

**Results:**

Following FB procedures, both WAD groups demonstrated generalized hypersensitivity to all sensory tests, decreased neck ROM and increased superficial muscle activity with the CCFT compared to controls (p < 0.05). There were no significant differences between WAD groups (all p > 0.05). Both WAD groups demonstrated psychological distress (GHQ-28; p < 0.05), moderate post-traumatic stress symptoms and pain catastrophization. The WAD_NR group also demonstrated increased medication intake and elevated PCS scores compared to the WAD_R group (p < 0.05).

**Conclusions:**

Chronic WAD responders and non-responders to FB procedures demonstrate a similar presentation of sensory disturbance, motor dysfunction and psychological distress. Higher levels of pain catastrophization and greater medication intake were the only factors found to differentiate these groups.

## Background

Whiplash associated disorders (WAD) are defined as the variety of symptoms arising from an initial whiplash injury usually as a result of a motor vehicle crash (MVC) [1]. The costs associated with WAD are substantial [1-3] with the majority of costs incurred by those individuals who transition to chronicity [4]. Approximately 50% of those injured report pain and disability at 12 months following the initial event [5].

There is now extensive evidence demonstrating marked physical and psychological changes in individuals with chronic WAD. These include sensory disturbances of widespread hypersensitivity [6-8] and hyperexcitable spinal cord reflexes [9,10] indicative of augmented central nervous system nociceptive processing (central sensitization). In addition, motor disturbances such as movement loss and altered muscle recruitment patterns have been clearly demonstrated [11-13]. Psychological distress (including affective disturbances, anxiety, depression and posttraumatic stress disorder symptoms) is also common in individuals with chronic WAD [14-16].

From a patho-anatomical perspective, the cervical facet joint is a common source of nociception in the neck region in individuals with chronic WAD [17-19]. Diagnosis of facet-mediated pain is possible through facet blocks (FB), be it intra-articular blocks (IAB) or comparative medial branch blocks (MBB) [20,21]. Effective treatment of facetogenic nociception has been demonstrated with radiofrequency neurotomy (RFN) [22], and may offer benefit to individuals who do not respond to conservative treatment following whiplash injury [23]. Recent synthesis of the literature and systematic reviews provide moderate levels of evidence that FBs effectively for determine of suitability for RFN [24-26]. Thus understanding the differences between those who do and do not respond to FB procedures is important.

Limited data is available describing individuals who do and do not respond to these procedures. Wasan et al. [27] showed that high comorbid pscychopathology was associated with less pain reduction following a single MBB for facet joint pain. However, this study did not include a wide range of measures reflecting the physical and psychological features consistently demonstrated to be present in chronic WAD. Some of the sensory, motor and psychological measures may influence responsiveness to these procedures. For example, central sensitization has been demonstrated to be a predictor of poor prognosis in individuals with musculoskeletal pain undergoing conservative treatment [28,29] and individuals undergoing surgery [30]; whilst catastrophization predicts poor response to painful procedures [31,32] and increased pain and disability ratings post surgery [33]. The presence of posttraumatic stress symptoms has also been demonstrated to result in more frequent pain and poorer prognosis in headache patients [34].

This preliminary study examined a sample of individuals who did and did not respond to FB as well as healthy controls to determine whether there were differences in their physical and psychological features once the effects of the blocks had abated and symptoms had returned. It was hypothesized that those who did not respond would have greater sensory, sensori-motor and psychological features than the responders and both groups would be different to the healthy controls. Such information is important to inform future predictive studies and ultimately the clinical selection of patients for FB procedures.

## Methods

### Design

This study was conducted in a tertiary spinal intervention centre in Calgary, Alberta, Canada. A cross-sectional study design was used to compare the clinical manifestations of two WAD groups: 1) WAD participants who responded to cervical facet joint double blockade and subsequently proceeded to, and were awaiting RFN (WAD_Responders); 2) WAD participants who failed to respond to cervical facet joint double blockade (WAD_Non-Responders); and a 3) healthy control group (HC). Individuals were admitted into the study at a time post-cervical facet joint injections when symptoms had returned and they reported were no different from those prior to receiving facet joint injections.

### Participants

#### Inclusion criteria

Consecutive participants were recruited from individuals aged 18–65 years with WAD Grade II [1] of a duration > 6 months post MVC who underwent scheduled cervical spine facet double block procedures (for predominant neck pain) (Intra-articular block - IAB and MBB). Those who responded (>50% relief of ‘neck’ pain) to both of the cervical facet double blockade procedures, and who were scheduled to progress to RFN entered as the WAD Responder (WAD_R) group. Individuals who did not respond to the initial cervical IAB procedure formed the WAD Non Responder (WAD_NR) group. Healthy control individuals with no previous history of neck pain, whiplash injury or recent treatment for musculoskeletal pain (within previous 2 years) were recruited from advertisements placed around the spinal intervention centre.

#### Exclusion criteria

Individuals were excluded from the study if they were classifiable as WAD Grade III or IV [1], or sustained a concussion or loss of consciousness as a result of the trauma. They were also excluded if their general health status prevented them from undergoing cervical facet double blockade procedure or RFN (e.g. central or peripheral neurological dysfunction such as stroke; peripheral vascular disease or coronary artery disease; pregnant, psychiatric history), or if they were not fluent in spoken or written English. Healthy controls were also excluded on these general health status criteria and all participants were excluded if they had sought recent treatment (previous two years) for a musculoskeletal condition or had received previous treatment for neck pain prior to the MVC.

All the participants were unpaid volunteers. Ethical clearance for this study was granted from the medical research ethics committee of the University of Queensland and the conjoint health research ethics board at the University of Calgary. All participants provided informed consent.

### Instrumentation

#### Motor measures

##### Range of motion

Active cervical range of motion (ROM) was measured using electromagnetic motion sensors (Fastrak, Polhemus, USA) [35]. One sensor was placed over the C7 spinous process and the other was attached to the top of a light skull cup, which was fitted to the participant’s head and firmly tightened, such that the second sensor sat on the vertex of the head. Three trials were performed in each direction (flexion, extension, left and right rotation) and the means of the three trials were used in analysis. A computer program was developed to convert the Euler angles into degrees of freedom of motion for the motion of the head (vertex) relative to the neck (C7 spinous process). The Fastrak has previously been used in trials of neck pain and whiplash participants [36] and has shown to be accurate within +/− 0.2 degrees [37].

##### Cranio-cervical flexion test

Surface EMG (Noraxon Tele Myo 900) was used to measure the activity of superficial neck flexor muscles (sternocleidomastoid - SCM) during the five incremental stages of the cranio-cervical flexion test (CCFT) as described by Jull [11]. The test was performed in supine and used a pressure biofeedback device (Stabilizer, Chattanooga, USA) placed sub-occipitally behind the neck to guide performance. It was inflated to a baseline of 20 mmHg and participants perform cranio-cervical flexion to increase the pressure by five progressive increments of 2 mmHg (22 mmHg-30 mmHg). Each pressure level was maintained for 10 s and participants rested for 15 s between each stage. Myoelectric signals were collected from the SCM muscles using Ag–AgCl electrodes (Noraxon, USA) in a bipolar configuration.

Electrodes were positioned along the lower one-third of the muscle bellies of the SCM [38]. Signals were amplified and filtered by a 500 Hz low pass filter (Noraxon TeleMyo 900, Scottsdale AZ) and sampled at 2000 Hz (National Instruments DAQ PCI-6221). EMG data were analyzed as follows: The maximum root mean squared (RMS) value was identified for each trace using a 1 s sliding window, incremented in 100 ms steps. RMS values were normalized for each participant, by dividing the 1 s maximum RMS from each level of the cranio-cervical flexion test by the 1 s maximum RMS during a standardized head lift. The baseline EMG data (RMS value) obtained at rest (20 mmHg) was subtracted from the measured EMG at each level of this test. The normalized RMS data for the left and right SCMs were averaged for analysis [11,36].

#### Quantitative sensory tests

##### Pressure pain thresholds

Pressure pain thresholds (PPTs) were measured using a pressure algometer (Somedic AB, Farsta, Sweden). The probe size was 1 cm^2^ and the rate of application was set at 40 kPa/sec. PPTs were measured over the articular pillars of C5/6 bilaterally (which is the most common facet joint involved in neck pain, (not involving headaches) following whiplash trauma); over the median nerve trunks anterior to the elbow bilaterally, and at a bilateral remote site (upper one third of the muscle belly of tibialis anterior) as previously described in investigations of chronic WAD [8]. The participants were requested to push a button when the sensation first became painful. Triplicate recordings were taken at each site and the mean value for each site used in the analysis.

##### Thermal pain thresholds

Thermal pain thresholds were measured bilaterally over the cervical spine using the TSA II Neurosensory Analyzer (Medoc Advanced Medical Systems; Minneapolis, MN, USA). The thermode was placed over the skin of the mid cervical region and preset to 32°C, with the rate of temperature change being 1°C per second. To identify cold pain thresholds (CPT) and heat pain thresholds (HPT), participants were asked to push a switch when the cold or warm sensation first became painful [39]. Triplicate recordings were taken at each site and the mean value for each site used in the analysis.

##### Brachial plexus provocation test

The brachial plexus provocation test (BPPT) was performed as described previously and in the following sequence: gentle shoulder girdle depression, glenohumeral abduction and external rotation in the coronal plane, forearm supination, wrist and finger extension, and elbow extension [40]. The range of elbow extension was measured at the participants’ pain threshold using a standard goniometer aligned along the mid humeral shaft, medial epicondyle, and ulnar styloid [41]. If the participant did not experience pain, the test was continued until end of available range.

##### Nociceptive flexion reflex

The nociceptive flexion reflex (NFR) is a polysynaptic spinal withdrawal reflex that is elicited following activation of nociceptive A-delta afferents [42]. It was performed via electrical stimulation through bipolar surface Ag/AgCl-electrodes (inter electrode distance approximately 2 cm), which were placed just distal to the left lateral malleolus of the ankle (innervation area of the sural nerve). EMG reflex responses to electrical stimulation were recorded from the middle of the biceps femoris and the (Ag/AgCl-electrodes). The participant lay prone and a wedge was placed under the ankle to obtain 30 degrees knee flexion. The EMG signal was amplified and low-pass filtered 0-500 Hz by a Multichannel EMG (Noraxon, Scottsdale AZ). Stimulation and recording was controlled and analyzed with custom software developed specifically for this test. A 25 ms, train-of-five, 1 ms, square-wave impulse (perceived as a single stimulus), was delivered by a computer-controlled constant current stimulator (Digitimer DS7A, England).

The current intensity was increased from 2 mA in steps of 2 mA until a reflex was elicited. The program delivered the impulses at random time intervals, so that the participants were not aware of when the stimulus was going to be applied. In this way, voluntary muscle contraction due to stimulus anticipation was avoided. A reflex response was defined using the standardized peak (NFR interval peak z score) EMG activity from biceps femoris as recommended [43]. The NFR Interval Peak z score is the NFR interval peak (EMG activity 90 to 150 ms post-stimulation interval)—baseline mean (60 ms before stimulation)/baseline SD. Rhudy and France [44], suggest a NFR interval peak z score of greater that 10.32 be used to define a reflex response. The 90 to 150 ms interval was chosen as it avoids possible contamination by low threshold cutaneous flexor reflex, startle reactions, and voluntary movements [44]. The current intensity required to elicit a reflex response was defined as the NFR threshold.

#### Questionnaires

Baseline measures included a description of symptoms, symptom dominance (unilateral or bilateral) and severity, crash parameters, treatments since the crash, compensation status, list of medications and demographic variables including gender, age, marital status, employment status, education level and duration of neck pain as per a standard clinical examination.

A single item visual analogue scale (VAS: 0-10 cm) was used to measure the participants’ pain intensity in the cervical spine with (0) described as ‘No Pain’ and (10) as ‘Worst Pain Imaginable’.

Self-reported pain and disability was measured in whiplash participants with the Neck Disability Index (NDI) [45]. The NDI consists of 10 items addressing functional activities such as personal care, lifting, reading, work, driving, sleeping, and recreational activities and also pain intensity, concentration, and headache which are rated from no disability (0) to total disability (5). The overall score (out of 100) is calculated by totalling the responses of each individual item and multiplying by 2. A higher score indicates greater pain and disability. It is the questionnaire most utilized in WAD research [46].

The s-LANSS is a validated self-report version of the Leeds Assessment of Neuropathic Symptoms and Signs pain scale [47]. It consists of seven items and includes two self-examination items. A score of 12 or greater indicates pain of a predominantly neuropathic nature. It has been used in previous WAD research [48].

All participants completed the General Health Questionnaire 28 (GHQ-28) [49] as a measure of general psychological distress. The General Health Questionnaire-28 (GHQ-28) is a 28-item measure of emotional distress in medical settings that is divided into 4 subscales: somatic symptoms (items 1 to 7), anxiety/insomnia (items 8 to 14), social dysfunction (items 15 to 21), and severe depression (items 22 to 28). Each item has a 4-point rating scale ranging from (0) to (3). The total scores can be used as a measure of psychological distress, with a higher score (>23/24) indicating greater distress. The GHQ-28 has been used in previous research of WAD [15,50].

The Posttraumatic Diagnostic Scale (PDS) [51] was included to assess symptom severity according to the Diagnostic and Statistical Manual of Mental Disorders (fourth edition, text revision; *DSM– IV–TR*) diagnostic criteria for post-traumatic stress disorders (PTSD). For every item, the frequency of the 17 PTSD symptoms within 1 week is assessed on a 4-point Likert scale, ranging from 0 (never) to 3 (daily). The items referred to a 1-month period prior to the study period. A total symptom severity score (ranging from 0 to 51) is derived with larger scores indicating greater symptom severity. The original PDS demonstrated high internal consistency and good stability and appeared to be a valid instrument for the assessment of PTSD in survivors of various traumatic events inclusive of motor vehicle crashes [52,53].

Pain catastrophizing was evaluated using the Pain Catastrophizing Scale (PCS) [31]. This is a 13-item questionnaire that describes various thoughts and feelings that individuals can experience when they are in pain, and requires participants to reflect on past pain experiences and to indicate the degree to which each of the items applied to them. Each item has a 5-point rating scale ranging from (0) not at all to (4) all the time and scores provide a total for the PCS. A total “cut-off score” of 30 reflects that an individual has clinically relevant pain catastrophization [54].

In both WAD groups, the following measures were completed: VAS, NDI, s-LANSS, GHQ-28, PDS and PCS. In the HC group, only the GHQ-28 questionnaire was completed.

### Procedures

#### Patient screening and participant group allocation

The referring physician nominated the spinal level and side of the facet joint block based on the individuals’ clinical presentation which the interventional radiologist reconfirmed based on clinical findings, including established pain maps [55]. Patients underwent a diagnostic IAB. A 25-gauge spinal needle was advanced under fluoroscopic guidance, into the target facet joint with the individual in the prone position. A small amount of nonionic contrast (0.5 cc of Omnipaque 300® Amerslan Health, Oakville, ON, Canada) was used to confirm needle position. Subsequently, an injection of 0.5 cc of local anaesthetic (1% Bupivicaine; AstraZeneca, Mississauga, ON, Canada), and 0.5 cc of corticosteroid (Celestone; Celestone Soluspan®, Schering, Pointe-Claire, Quebec, Canada) was made into the target facet joint, until resistance was felt. If the contrast-medication mixture leaked from the joint, this was noted in the procedure report, as diagnostic specificity may be affected.

During the post-injection follow-up period (a minimum of two hours), participants who reported a decrease in ‘neck’ pain intensity of at least 50%, and concurrently reported a significant improvement in symptoms (of their ‘main’ and familiar pain) for the duration of the anaesthetic were determined to have responded to the IAB. If pain returned within the following days or weeks, they underwent a second diagnostic cervical facet joint block, a confirmatory MBB as advocated for the diagnosis of facet joint pain [20,56,57]. The MBBs were only performed at a time when the familiar pain returned. If an individual had prolonged relief of pain (generally > 3 months) following the IAB, then confirmatory MBBs were not performed. As these individuals did not receive subsequent MBB, a diagnosis of ‘facet pain’ could not be confirmed, and these individuals were not included in the study. The MBB involved the placement of a 25-gauge spinal needle, under fluoroscopic guidance, onto the medial branch of the dorsal ramus as it courses over the waist of the articular pillar at each spinal level. An injection of nonionic contrast material (0.5 cc of Omnipaque 300® Amerslan Health, Oakville, ON, Canada) was made to confirm needle position. Subsequently, 0.5 cc of 2% Lidocaine (AstraZeneca, Mississauga, ON, Canada), was injected onto the medial branch of the dorsal ramus. Both medial branches to the target facet joint were anaesthetized in order to effectively anaesthetize the joint [57].

For the purposes of this current study, the patient was assigned to the WAD_R group if they had a successful response to the MBB (>50% relief of neck pain) for the duration of the anaesthetic and agreed to participate in the study.

If the first IAB block was negative, investigations were either terminated or initiated at another segmental level that might reasonably have been responsible for the pain. In this manner, blocks were continued until all such possible levels either proved negative or until a positive response was encountered. This practice was recently recommended to assist with diagnostic accuracy and in an attempt to reduce the false negative rate [58]. Thus, these patients underwent procedures directed at their familiar pain, such that if their predominant symptom was ‘upper’ neck pain, the upper cervical facet joints (C2-4) were injected, whilst if their predominant symptom was ‘lower’ neck pain, then the lower cervical facet joints (C4-7) were injected [55]. If an individual had ‘upper’ and ‘lower’ neck pain, or mid-level neck pain, then all facet joints were injected (C2-7) to rule out the presence of facet-mediated pain. A negative response was defined as no relief of pain with any procedure. These individuals were subsequently assigned to the WAD_NR group.

Clinically, this diagnostic pathway is used prior to consideration for RFN [59]. There is some discussion in the literature regarding the optimum percentage of pain relief an individual should experience to fulfill the operational definition of a ‘successful response’ [60,61]. To our knowledge, only one study has investigated this response in the cervical spine, with no significant difference in outcomes reported in patients with either 50% or 80% pain relief after their diagnostic block [60]. While 80% relief of pain is cited as the reference standard for research purposes [62], many clinicians feel that 50% relief is clinically significant [63]. From a practical perspective, individuals with this response were historically noted in our clinic to successfully respond to future RFN.

#### Study measurements

Measurements occurred approximately one month following the ‘failed’ IAB (for the WAD_NR group participants), or ‘successful’ MBB (for the WAD_R participants). All participants attended the research laboratory at a time point following procedures whereby their ‘familiar’ pain had returned to the level reported prior to receiving the procedures. On arrival at the research laboratory, all participants underwent an examination by an experienced physiotherapist with postgraduate qualifications to reconfirm their eligibility before inclusion in the study. Participants were given a written description of the study procedures and informed consent was gained before proceeding to the questionnaires and testing. Familiarization sessions were performed for each measure. Participants practiced all movements or instructions until they felt comfortable to proceed.

After completion of the questionnaires, a standard protocol was used for the order of tests [64]. The participants were seated, the Fastrak sensors applied and ROM was measured. The participants were then positioned supine, EMG electrodes were applied, and the CCFT was performed. For all of the following bilateral tests, the left side was measured first. PPTs were measured in the following order: tibialis anterior, median nerves and C5/6. Thermal pain thresholds were then measured over the cervical spine, HPTs followed by CPTs; followed by the BPPT. The NFR was the final testing procedure. The same examiner tested all participants. No feedback or cues were given to the participants regarding their performance on any tests.

### Data analysis

Data were analyzed with Stata 9.0 statistical software. Based on our previous research [59], our statistical calculations indicated that this study required 26 participants (with 80% power at 5% level of significance) to adequately detect a minimally clinically important difference for the following physical measures: change in Tibialis Anterior PPT, change in CPT, or change in NFR threshold.

Assumptions of normality, nonmulticollinearity, and homoscedasticity were tested through examination of histograms, box plot graphs, correlation matrices, and a plot of predicted to residual values, respectively. If the data were not normally distributed, transformation of the data was applied to interval data. PPT, NFR, CCFT and BPPT data required log transformation. If normality was not achieved following transformation (CPT, HPT), medians and interquartile ranges were generated. The Wilcoxon matched-pairs signed-rank test was used initially used to determine within participant side to side differences and followed by the exploratory analysis for all the measures and in all groups. Where no side-to-side differences existed (CPT, BPPT), the data from each side was compiled and averaged, with the mean compiled data used for analysis. Where ‘side-to-side’ differences existed within groups for various measures, the mean measure of each ‘side’ was analyzed between groups. There was a significant side to side difference in the WAD_R group for HPT (p = 0.007). There was also a significant difference in PPT measurements between right and left cervical spine (p = 0.001) and Tibialis Anterior (p = 0.04) Pin the HC group (p = 0.001). As a result, group analyses for these measures were performed for each individual test site performed.

Chi-squared analysis was utilized to determine if there was a difference in proportions of individuals in the WAD groups with respect to compensation status, employment, education, marital status, number of bodily symptoms and above threshold scores for GHQ-28, PCS, PDS and s-LANSS.

Multivariate analysis of variance (MANOVA) was performed to investigate the effect of group (WAD_R, WAD_NR or HC) on the following log-transformed measures: PPT and CCFT, and normally distributed ROM. One way analysis of variance (ANOVA) tests were used for log-transformed BPPT and NFR measures. Where there was a significant group difference, post hoc tests of simple effects were performed to determine where these differences occurred. Non-parametric Kruskal-Wallis rank tests were used to determine any significant group differences for CPT and HPT measures. Non-parametric tests were used to analyze group differences in the following ordinal-scored questionnaires where homoscedasticity was present, but normality was not achieved (GHQ-28: Kruskal-Wallis; PCS, PDS and s-LANSS: Mann–Whitney). Differences between groups were analysed using a priori contrasts. Significance level was set at 0.05 with Bonferroni adjustments used (for normally distributed data); and the Least Significant Difference (LSD) in ranks was calculated if significance was achieved using the Kruskal-Wallis rank test [65].

## Results

### Participants

**Figure 1:**
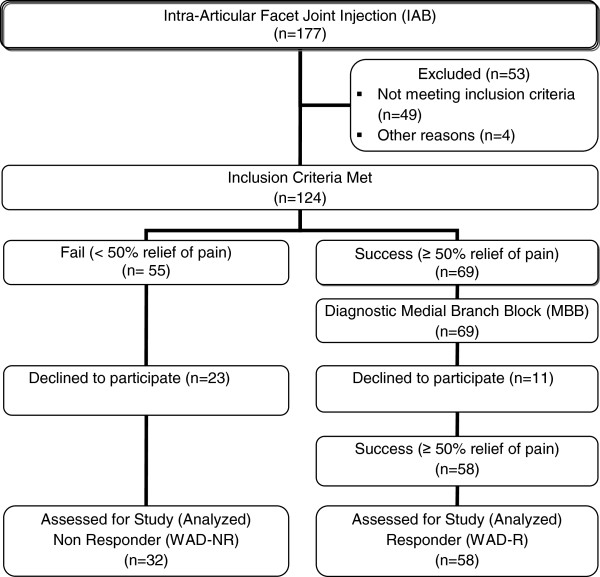
Flow of participants through the study.

Ninety individuals undergoing IAB injections fulfilled the inclusion criteria and agreed to participate (32 males, 58 females, mean age 45.1 +/− 10.6 (SD) years). Fifty-eight individuals responded to the cervical facet double block procedure (IAB and MBB: 18 males, 40 females, mean age 44.9 +/− 11.1 years) and formed the WAD_R group. The C5/6 facet joint was the most common symptomatic joint either alone or in combination with another joint (Table 1). Thirty-two individuals did not respond to the IAB (14 males, 18 females, mean age 45.4 +/− 9.7 years) and formed the WAD_NR group. Thirty healthy individuals (9 males, 21 females, mean age 44.2 +/− 9.7 years) formed the HC group. Figure 1 demonstrates the flow of participants through the study.

**Table 1:** The prevalence of cervical joints injected (n = 90)

**Group (n)**	**C2/3 (%)**	**C3/4 (%)**	**C4/5 (%)**	**C5/6 (%)**	**C6/7 (%)**
WAD_R (58)	41	47	33	48	28
WAD_NR (32)	33	34	38	64	42

**Legend:** WAD_R = WAD Responders; WAD_NR = WAD Non-Responders.

The median [range] duration of symptoms post whiplash was 42 [9 – 195] months. All participants received initial treatment following the MVC, consisting mainly of pharmaceutics (a combination of various medications such as over-the-counter analgesics, anti-inflammatories, anti-depressants, opioids and anti-convulsants – Table 2) and various therapeutic treatments, including physiotherapy, massage therapy, acupuncture, and chiropractic. A greater proportion of WAD_NR individuals were taking each class of medication. Thirty-four participants in the WAD_R group (59%) and 16 in the WAD_NR group (50%) were receiving conservative treatment at the time of participation in the study.

**Table 2:** Medication use at intake of each whiplash participant

**Medication**	**WAD_R (%)**	**WAD_NR (%)**
Anti-Inflammatory	38	47
Over-the-counter Analgesics	29	34
Anti-Convulsant	17	19
Opioid	16	25
Muscle Relaxant	9	16
Anti-Depressant (SNRI)	7	9
Anti-Depressant (TCA)	7	16

**Legend:** WAD_R = WAD Responders; WAD_NR = WAD Non Responders; n = number; SNRI = Serotonin-Norepinephrine Reuptake Inhibitors; TCA = Tricyclic Antidepressants.

Table 3 presents the demographic, pain and disability characteristics for the groups. There were no significant differences in gender or age between the three groups (p > 0.2) and no differences in pain (VAS and s-LANSS) and disability (NDI) scores between the WAD groups (p > 0.1). Twenty-nine participants in the WAD_R group (50%) and 19 participants in the WAD_NR group (59%) were involved in ongoing compensation claims but this difference was not significant (χ^2^ = 0.73, 1 d.f., p = 0.39). Likewise there were no differences between the WAD groups with respect to the presence of other bodily pain (number of symptoms), education levels, marriage or employment status (p > 0.1). WAD groups did not differ to the healthy control group in relation to education levels, marriage and employment status (p > 0.1).

**Table 3:** The demographic characteristics of subject groups

**Group (n)**	**Gender (% F/M)**	**Age mean yrs (+/− SD)**	**Duration of symptoms median mths [Range]**	**VAS mean (+/− SD) (0-100 mm)**	**NDI mean (+/− SD) (%)**	**s-LANSS median [IQR]**
WAD_R (58)	69%	44.3 (10.4)	44 [9 – 195]	59 (18)	42 (15)	11 [8-17]
WAD_NR (32)	56%	45.4 (9.7)	34 [10 – 190]	63 (19)	47 (14)	13 [8-16]
HC (30)	70%	44.2 (9.7)				

**Legend:** n = number; F = female; M = male; SD = Standard Deviation; VAS = Visual Analogue Scale; NDI = Neck Disability Index; s-LANSS = self administered Leeds Assessment of Neuropathic Symptoms and Signs; IQR = InterQuartile Range; WAD_R = WAD Responders; WAD_NR = WAD Non Responders.

### Physical measures

#### Pressure pain thresholds

MANOVA revealed a significant difference between the three groups at all test sites (neck, median nerve and tibialis anterior: F_12,224_ = 4.71, p < 0.001; Table 4). Post-hoc tests showed that both whiplash groups demonstrated lower PPTs at all sites compared with the healthy control group (F_6,112_ = 9.53, p < 0.001). There were no significant differences between the whiplash groups (F_6,112_ = 0.71, p = 0.64).

**Table 4:** **Median [Interquarterile range] scores and *p *** **values for sensory measures**

**Group (n)**	**PPT_Cx (kPa) Med [IQR]**		**PPT_Med (kPa) Med [IQR]**		**PPT_TibAnt (kPa) Med [IQR]**		**CPT (°C) Med [IQR]**	**HPT (°C) Med [IQR]**		**BPPT (°elb ext) Med [IQR]**	**NFR (mA) Med [IQR]**
	**L**	**R**	**L**	**R**	**L**	**R**		**L**	**R**		
HC (30)	327 [246-410]	363 [302-466]	336 [286-429]	377 [305-518]	531 [471-692]	575 [472-743]	3.5 [0-8.1]	47.5 [45.7-48.8]	47.4 [45.9-48.7]	**3** [0-9]	21 [10-38]
WAD_R (58)	171^*^ [141-238]	185^*^ [139-230]	226^*^ [179-284]	249^*^ [186-292]	315^*^ [254-368]	337^*^ [284-424]	**19.7**^**‡**^ [11.3-25.4]	**42.7**^**‡ **^[40.2-47.4]	**41.7**^**‡**^ [39.4-45.6]	30^*^ [18-40]	**12**^**‡**^ [8-18]
WAD_NR (32)	166^*^ [120-229]	149^*^ [110-257]	231* [177-285]	229^*^ [166-288]	322^*^ [252-425]	338^*^ [237-471]	**17.4**^‡^ [6.4-26.4]	**44.2**^**‡**^ [40.2-47.0]	**42.6**^**‡**^ [37.9-46.6]	34^*^ [24-44]	**12**^**‡**^ [8-16]
MANOVA	p<0.001		p<0.001		p<0.001		Kruskal-Wallis: p<0.001	Kruskall-Wallis: p<0.001		ANOVA: p<0.001	ANOVA: p<0.01

**Legend:** PPT = Pressure Pain Threshold; kPa = kilopascals; Cx = Cervical; Med = Median Nerve; TibAnt = Tibialis Anterior; CPT = Cold Pain Threshold; HPT = Heat Pain Threshold; BPPT = Brachial Plexus Provocation Test; elb ext = elbow extension range of motion; NFR = Nociceptive Flexion Reflex; mA = milliamps; L = Left; R = Right; HC = Healthy Control; WAD_R = WAD Responders; WAD_NR = WAD Non Responders; *p < 0.001; ‡p < 0.05.

#### Thermal pain thresholds

Kruskal-Wallis Rank tests revealed a significant difference between the mean ranks of thermal thresholds per individual (for both cold pain threshold (CPT) and heat pain threshold (HPT) measurements) among the three groups (H > 18.9, 2 d.f., p < 0.001; Table 4). Post hoc testing revealed that both whiplash groups demonstrated elevated CPT (LSD > 30.2, p < 0.05) and reduced HPT (LSD > 30.7, p < 0.05) when compared to the healthy control group. There were no differences between the two whiplash groups for either cold pain thresholds (LSD = 5.2, p > 0.05) or heat pain thresholds on either side of the neck (LSD < 2.3, p > 0.05).

#### Brachial plexus pain provocation test

ANOVA revealed significant differences between the three groups for elbow extension ROM (F_2,100_ = 27.72, p < 0.001; Table 4). Post-hoc tests showed that the WAD_R and WAD_NR groups demonstrated restricted elbow extension ROM when compared to healthy controls (p < 0.001). There were no significant differences between the whiplash groups (p = 0.87).

#### Nociceptive flexion reflex

ANOVA revealed significant differences between the three groups for NFR threshold (F_2,116_ = 5.52, p < 0.01; Table 4). Post-hoc tests showed that the whiplash groups required less current to elicit the reflex than the healthy control subjects (p < 0.05). There were no significant differences between the two whiplash groups (p = 1.00).

#### Range of motion

**Figure 2:**
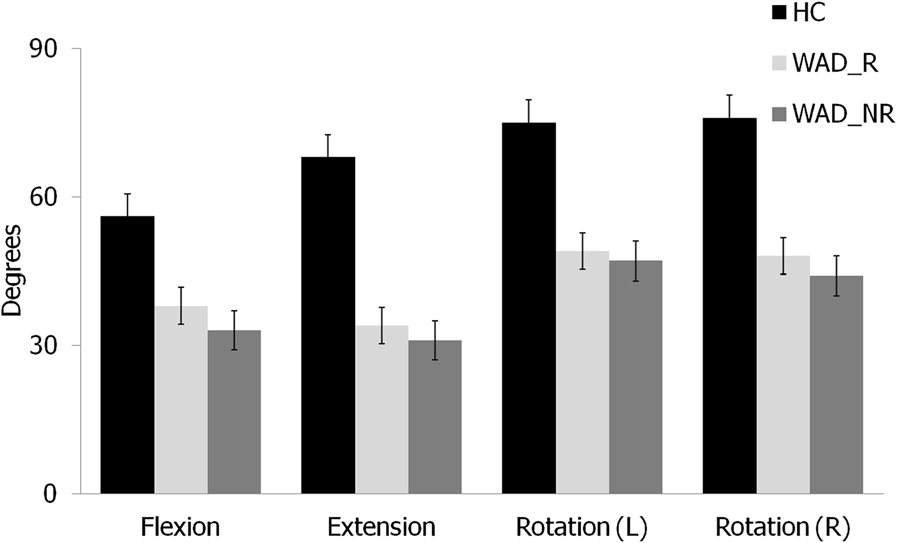
**Comparison of cervical ROM between groups.** ROM = Range of Motion; HC = Healthy Controls; WAD_R = WAD Responders; WAD_NR = WAD Non-Responders; (L) = Left; (R) = Right.

MANOVA revealed significant differences between the three groups in ROM (F_8,228_ = 22,88, p < 0.001). Post-hoc tests revealed that the two whiplash groups demonstrated significant less ROM compared to the healthy control subjects (F_4,114_ = 62.29, p < 0.001). There were no statistically significant differences in ROM in any direction between the two whiplash groups (F_4,114_ = 1.09, p = 0.37; Figure 2).

#### Cranio-cervical flexion test

**Figure 3:**
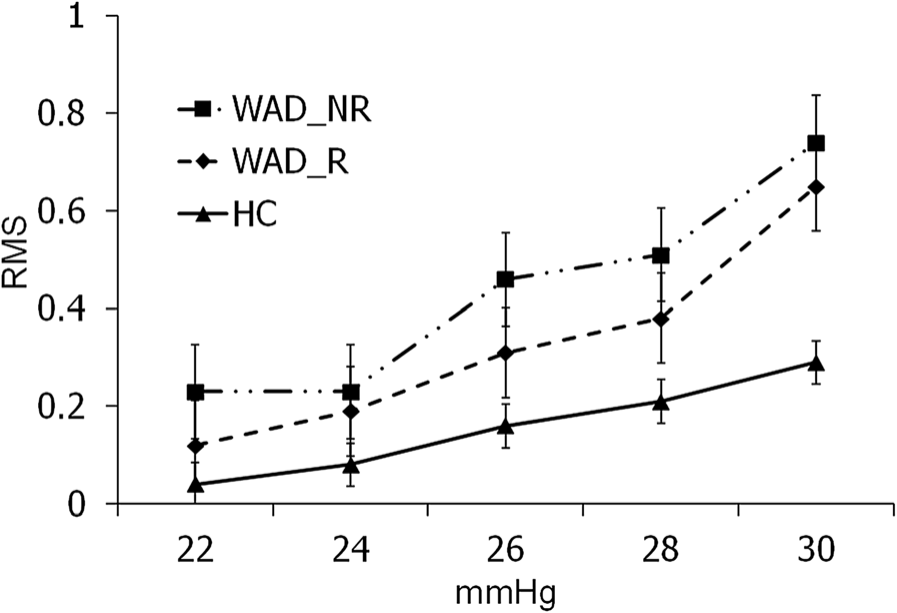
**Cranio-cervical flexion test performance across groups.** RMS = Root Mean Square; HC = Healthy Controls; WAD_R = WAD Responders; WAD_NR = WAD Non-Responders.

MANOVA revealed significant differences between the three groups for EMG activity of the superficial neck muscles at all stages of the cranio-cervical flexion test (CCFT: F_10,224_ = 3.34, p < 0.001). Post-hoc tests revealed significant differences between the whiplash and healthy control groups (F_5,112_ = 5.98, p < 0.001). No statistically significant differences existed between the two whiplash groups (F_5,112_ = 1.7, p = 0.14; Figure 3).

### Psychological measures

The median scores, interquartile ranges and proportion of participants exceeding threshold scores for GHQ-28, PCS, and PDS for the three groups are presented in Table 5.

**Table 5:** Median [Interquartile range] scores of each group for psychological measures

**Group (n)**	**GHQ-28% ≥23**	**Score [IQR]**	**PCS % ≥30**	**Score [IQR]**	**PDS % met criteria probable PTSD**	**Severity score [IQR]**
WAD_R (58)	64%	24^*^ [19-32]	16%	15 [7-23]	29%	7 [2-13]
WAD_NR (32)	66%	28^*^ [21-41]	50%^*^	**30**^**‡**^ [13-39]	44%	12 [5-20]
HC (30)	7%	14 [10-16]				

**Legend:** GHQ = General Health Questionnaire; PCS = Pain Catastrophization Scale; PDS = Post Traumatic Stress Diagnostic Scale; WAD_R = WAD Responders; WAD_NR = WAD Non Responders; HC = Healthy Controls; *p < 0.001; ‡p < 0.01.

Both whiplash groups demonstrated significantly higher GHQ-28 total scores (H = 38.2, 2 d.f., p < 0.001) compared to healthy controls. There was also a significant greater proportion of whiplash individuals with generalized psychological distress (GHQ-28 > 23/24, p < 0.001) - 64% of WAD_R individuals and 66% of WAD_NR individuals scored above threshold (>23/24), compared to 7% of controls. There was no significant difference in psychological distress between the two whiplash groups (LSD = 8.1, p > 0.05).

There was no difference in the proportion of individuals in the two whiplash groups fulfilling the criteria for PTSD (χ^2^ = 1.90, 1 d.f., p = 0.168) with 29% of WAD_R and 44% of WAD_NR group meeting the PDS criteria. The results also suggest that there is no statistically significant difference between the post traumatic stress severity scores of the two whiplash groups (z = 1.69, p = 0.09).

There was a significantly greater proportion (χ^2^ = 12.22, 1 d.f., p < 0.001) in the WAD_NR group (50%) with elevated Pain Catastrophization scores (PCS ≥ 30) [54], compared to 16% in the WAD_R group. Significantly higher PCS scores were also reported by the WAD_NR individuals (z = 2.7, p = 0.006).

## Discussion

Our hypothesis, that individuals with chronic WAD who did not respond to FB procedures (WAD_NR), would have greater sensory, sensori-motor and psychological features than responders (WAD_R) was largely rejected; with few between group differences demonstrated. However, the results did reveal that both WAD groups were different to the healthy controls (HC). Possible reasons for these findings are discussed.

Our participants with WAD presented similar profiles to previous studies and support findings that chronic WAD demonstrates a complex clinical presentation including sensory hypersensitivity, sensori-motor dysfunction and psychological distress [66,67]. Pain and disability levels were comparable to other patients undergoing MBB [17,18,22,68]. Some individuals reported an extensive duration of neck pain, and although the literature indicates the episodic nature of neck pain over time [69], all individuals reported that their symptoms were attributable to an original MVC. In concert with other studies, our participants reported lower pain thresholds to pressure and thermal stimuli [70-72] heightened responses bilaterally to BPPT [73,74], reduced NFR thresholds [9,10], decreased cervical ROM [35,36,75] and impaired control of cranio-cervical flexion [11,36,76]. Our healthy control data were likewise similar to that previously reported [11,77,78]. The psychological profile of our whiplash participants is also consistent, with high levels of psychological distress [15,16], moderate post traumatic stress symptoms [79] and levels of pain catastrophizing [80] evident.

The presence of sensory hypersensitivity likely reflects central nervous system hyperexcitability [81,82] indicating that similar nociceptive processes underlie the conditions of both groups. Higher levels of pain and disability have been associated with the presence of these sensory features in WAD [8] and 82% of our participants reported moderate to severe levels of pain related disability. Thus, it could be expected that sensory hypersensitivity would be a feature of both groups irrespective of responsiveness to the joint block techniques. There were also no differences in measures of motor function between the two whiplash groups. Loss of neck movement and impaired performance on the CCFT are also features of other neck pain conditions including non-traumatic idiopathic neck pain and cervicogenic headache [35,83]. Whilst there may be some relationship with levels of pain and disability [36], the uniform presence of motor dysfunction across neck pain conditions suggest that our findings are not unexpected.

Levels of psychological distress as measured with the GHQ-28 were no different between our whiplash groups and are not surprising considering the levels of pain and disability reported by the participants. Whilst not reaching statistical significance, a greater proportion of non-responders fulfilled the criteria for a PTSD diagnosis on the PDS questionnaire (44% of non-responders versus 29% of responders) and reported higher symptom severity levels. The lack of statistical significance may be a consequence of the sample size of the study and this factor requires further investigation, especially given recent studies that demonstrate a relationship between PTSD, and pain/disability in WAD [84-86].

There was one notable difference between the two whiplash groups. Higher levels of pain catastrophization were demonstrated in the WAD_NR group. Catastrophization has been associated with enhanced pain reports, concurrent disability [80,87] and lower pain threshold/tolerance levels, but is not significantly related to nociceptive flexion reflex (NFR) threshold in healthy and clinical pain samples [10,88]. Sullivan et al. [31] reported that higher levels of catastrophization predicted higher levels of pain following medical procedures, such that these individuals may actually be less responsive to invasive interventions. It is possible that the higher levels of catastrophization and tendency towards higher psychological distress and post traumatic stress symptoms observed in the WAD_NR group may have contributed to the lack of response to the facet joint injection. The exact mechanisms responsible for this lack of responsiveness require further investigation, but may even include diminished placebo responses, where individuals may not ‘believe’ in the blocks or invasive procedures. Alternately, the higher PCS scores in our non-responder group may be a consequence of the study methodology. PCS scores were obtained *following* diagnostic facet joint procedures in both whiplash groups. It is possible that a lack of response may increase levels of catastrophization.

The WAD_NR group reported greater medication intake than the responder group and this was the case for all medication types. Given that pain and disability levels were no different between the groups, it could suggest that higher levels of catastrophization may explain the need for increased medication; or alternately, the lack of effectiveness of medication in reducing pain and disability may result in higher levels of catastrophization. There is some data available to support the initial claim suggesting that catastrophization is associated with greater medication intake [33]. However, this requires further investigation.

The few differences found between the two groups in both physical and psychological measures would seem to indicate that similar processes are contributing to the clinical presentation, regardless of whether or not facet joint nociception is involved. It is possible that the WAD_NR group may have nociception arising from other structures. Cadaver and biomechanical studies indicate that various cervical spine structures can be potentially injured during whiplash trauma mechanisms and structures other than the cervical facet joints may be responsible for ongoing nociception [89-91]. However, it has also been proposed that factors other than peripheral nociception, for example physiological stress responses, can induce hyperalgesic responses and these may explain the presence of various symptoms in individuals with chronic WAD [92-94]. Future studies are currently underway to investigate the attenuation of the physical and psychological features of chronic WAD following modulation of facet joint nociception, to assist in understanding this relationship further.

Wasan et al. [27] previously demonstrated that psychiatric co-morbidity is associated with reduced pain reduction following MBB, however they utilized different scales (Hospital Anxiety and Depression Scale); focussing on symptoms of anxiety and depression whereas this current study evaluated psychological distress (GHQ) and post traumatic stress symptoms (PDS). It may be that affective/anxiety symptoms have a greater association with response to MBB. Additionally, symptoms may not be as important as actual diagnosis in predicting response to MBB. There was certainly a trend towards an increased proportion of PTSD diagnoses in the WAD_NR group that may be of significance in a larger study. Therefore, further investigation of psychological diagnoses, and the role of pain catastrophization and posttraumatic stress symptoms in outcomes following procedural interventions would be indicated.

Consideration must be given to the diagnostic facet joint blockade procedures and ‘cut-points’ used in our study. The use of comparative local anaesthetic blocks or placebo blocks has been advocated to guard against false positive responses [57]. In this study, two diagnostic injection procedures were used, IAB followed by MBB. This combination of diagnostic techniques possesses a similar construct to comparative MBB’s, with individuals reporting relief of their predominant pain for the duration of the anaesthetic. Target specificity was ensured with each procedure by the use of radiographic confirmation of contrast medium (without note of radiate spread) to ensure needle location [95]. The responder patients in this study reported a consistent response to both procedures (50% or greater decrease in pain intensity).

Whilst placebo blocks are preferred for ensuring diagnostic accuracy in the cervical region [96], this was not possible at the clinic where our study was conducted. Therefore, whilst the approach used in our clinic was stringent, we cannot fully exclude a placebo effect in responders or a nocebo effect in non-responders. A lack of differences between the whiplash groups may have also resulted from the criterion standard utilized in our study for determining ‘success’ of the intervention. The clinic used in the study refers individuals for RFN if they report ‘greater than 50% relief of pain’ following confirmatory MBB. This cut-off may not be sufficiently sensitive to detect differences between the responder and non-responder groups. Eighty percent pain relief has been suggested for use in research studies [62], but our study was required to use 50% to adhere to the protocol required by the clinic involved. Of note, previous research has shown no difference in clinical outcomes following RFN when 50% versus 80% pain relief from FB was used as the criterion standard [60].

It was also noteworthy that more individuals who failed to respond to the MBB were lost to follow-up. As Figure 1 demonstrates, 23/55 (42%) people who did not respond to IAB were lost to follow-up, compared to only 11/69 (16%) of those who responded. Comparison of these individuals was not possible and the effects on the results are not known.

Another possible limitation of this study was that the measures performed in this study were performed by the study author, who was aware of the study hypotheses, however considerable care was made to avoid describing study aims to the participants during the study (and expectations of results were unknown given it was a descriptive study); however bias is possible when examiners are not blinded.

This study was a preliminary cross-sectional study to investigate any physical or psychological differences in a cohort of individuals with chronic WAD who did and did not respond to cervical FB procedures. The design has limitations, but the results serve to inform future predictive studies. Inclusion of the physical measures (i.e. sensory and motor measures) in future prospective studies, may be necessary for profiling patients, but is unlikely to be predictive of response. Our findings do suggest that a wider raft of psychological measures be explored, given some differences in these domains. In addition, the inclusion of measures such as locus of control, coping styles and expectations, may ultimately assist the clinical selection of patients for FB procedures.

## Conclusion

Individuals with chronic WAD who respond and who do not respond to facet joint injections display similar complex clinical manifestations involving sensory disturbances, motor dysfunction and psychological distress. The presence of high levels of pain catastrophization and post-traumatic stress symptoms requires further investigation to determine their roles in non-responsiveness to FB.

## Abbreviations

BPPT: Brachial plexus provocation test; CCFT: Cranio-cervical flexion test; CPT: Cold pain threshold; EMG: Electromyography; FB: Facet blocks; GHQ-28: General health questionnaire-28; HC: Healthy controls; HPT: Heat pain threshold; IAB: Intra-articular block; LSD: Least significant difference; M(ANOVA): Multivariate (analysis of variance); MBB: Medial branch block; MVC: Motor vehicle crash; NDI: Neck disability index; NFR: Nociceptive flexor reflex; PCS: Pain catastrophizing scale; PDS: Posttraumatic stress diagnostic scale; PPT: Pressure pain threshold; PTSD: Posttraumatic stress disorder; RFN: Radiofrequency neurotomy; RMS: Root mean squared; ROM: Cervical range of movement; SCM: Sternocleidomastoid; s-LANSS: Self report Leeds Assessment of Neuropathic Symptoms and Signs; VAS: Visual analogue scale; WAD: Whiplash associated disorders; WAD_R: WAD responders; WAD_NR: WAD non-responders.

## Competing interests

The authors declare that they have no competing interests.

## Authors’ contributions

AS: research design, data collection and statistical analysis, manuscript preparation and revision. GJ: research design, manuscript preparation and revision. GS: research design, manuscript preparation and revision. AH: research design, manuscript preparation and revision. BF: research design, manuscript preparation and revision. MS: research design, data analysis, manuscript preparation and revision. All authors read and approved the final manuscript.

## Pre-publication history

The pre-publication history for this paper can be accessed here:

http://www.biomedcentral.com/1471-2474/14/313/prepub
